# Perpetual Futures for Stocks: The SpaceX Pre-IPO Market

**DOI:** 10.3390/e28090950

**Published:** 2026-08-24

**Authors:** Aditya Gupta, Nicholas G. Polson

**Affiliations:** 1 Stochastic Processes, New York, NY 10013, USA; 2 Booth School of Business, University of Chicago, Chicago, IL 60637, USA; ngp@chicagobooth.edu

**Keywords:** perpetual futures, funding rate, price discovery, no-arbitrage, martingale pricing, nonlinear filtering, stochastic volatility, time change, market segmentation, pre-IPO equity, generative Bayesian computation, G12, G13, G14, C11

## Abstract

Robert Shiller proposed perpetual futures in 1993 to create derivative markets for assets that are illiquid or whose price cannot be observed directly. Cryptocurrency markets later built the instrument under a different funding rule. We give a single no-arbitrage result that nests both designs: the perpetual price is the present value of a benchmark flow discounted at the funding rate, so the funding rule fixes both the benchmark and the discount. A random time change represents the price as the expected spot at the first event of a clock whose intensity is the funding rate. This yields the main structural result, that stochastic volatility moves the basis only through the carry, so a volatility risk premium, and not volatility itself, can break the peg. We then read price discovery as nonlinear filtering in which the funding rule is a feedback observer whose gain is the funding intensity and the peg the fixed point of a stochastic approximation, and we give a segmented market equilibrium under which the pre-listing premium is structural rather than behavioral. In the June 2026 SpaceX market, the last pre-listing closes were $172.84 on Hyperliquid and $170.82 on Binance, compared with the listed equity’s $185 close on 18 June and the $135 bookbuilt offer. Simulation matches the pricing results to their closed forms. Generative Bayesian computation recovers the funding intensity sharply but not the softness of the anchor.

## 1. Introduction

Derivative markets usually presuppose a liquid, observable spot against which a contract settles. Many economically important claims have no such spot: a single family home, a unit of human capital, a basket of macro risk, or the equity of a private firm. Shiller [1] proposed the perpetual future to reach these claims, settling a non-expiring contract each period against an observable flow rather than a price. The design lay mostly dormant until cryptocurrency venues rebuilt it with a different settlement, a funding payment keyed to the basis against a spot, and made it the dominant traded instrument in that market. In June 2026, the same instrument was used at equity scale for the first time, to trade SpaceX before and through its public listing.

This paper gives a unified treatment and applies it to that episode. First, a single no-arbitrage result, Proposition 1, shows any perpetual prices as the present value of a benchmark flow discounted at the funding rate, so the funding rule alone separates the Shiller design from the crypto design and the two are one instrument with two benchmarks. Second, Proposition 2 gives a random time change representation: the price is the expected spot at the first event of a clock whose intensity is the funding rate. Third, Corollary 3 shows that stochastic volatility leaves the peg intact unless it is priced into the carry, while Proposition 5 gives the resulting exact basis, the perpetual analogue of the futures-minus-forward adjustment. Fourth, we read price discovery as filtering and show in Section 8 and Section 9 that the funding rule is a feedback observer, the price a Doob martingale of the latent value, and the peg a Bayesian fixed point. Fifth, Proposition 6 gives a segmented market equilibrium for the basis. Sixth, Section 13 verifies the pricing results against closed forms on simulated data, validates the estimation procedure, and calibrates the filtering model. Seventh, we work the SpaceX example. We write under a fixed risk-neutral measure Q throughout the pricing sections and pass to the physical measure P only when reading the premium.

**Relation to the literature.** The paper draws three pieces of literature together: The first is the work of Shiller [1,2] on cash-settled markets for unobservable assets, of which the perpetual future is one instrument. The second is the no-arbitrage analysis of crypto perpetuals by He, Manela, Ross and von Wachter [3], Angeris, Chitra, Evans and Lorig [4], and Ackerer, Hugonnier and Jermann [5] with the limits to cross-market arbitrage emphasized by Makarov and Schoar [6]. The third is the filtering and martingale theory of price formation, from the work of Kalman [7] and the martingale pricing of Harrison and Kreps [8] to generative Bayesian computation [9].

Our position relative to the second of these should be stated precisely, since it is the closest antecedent. He, Manela, Ross and von Wachter [3] derive the crypto perpetual as a rolling contract pegged to an observable spot and characterize the funding rate that enforces the peg. Ackerer, Hugonnier and Jermann [5] study the frictional case and give sharp deviation bounds around it. Both take the spot as given and observable, which is the case that matters for a liquid cryptocurrency. Three things are new here: First, we do not fix the benchmark; Proposition 1 holds for an arbitrary adapted settlement pair, and the Shiller dividend design and the crypto basis design appear as two choices of that pair rather than as separate instruments, which is what lets us treat them in one framework. Second, we allow the underlying to be latent. Once the spot is an estimate rather than a transaction price, the funding rule ceases to be an arbitrage mechanism and becomes an inference mechanism, and Section 8 and Section 9 identify the funding intensity with an observer gain and the peg with a Bayesian fixed point. That reading has no counterpart in the existing pricing literature, which does not need it because a liquid spot leaves nothing to infer. Third, the random time change representation of Proposition 2 gives the volatility result of Corollary 3 as a one-line optional sampling argument, where the existing treatments proceed by direct computation under specific dynamics. The unifying claim is that the funding rate is simultaneously a discount rate, a clock, an observer gain, and a learning rate.

**Terminology.** Three terms recur and are defined here on first use: A *hard spot* is a contemporaneously observable transaction price in the underlying, against which the basis trade of Proposition 3 can be executed. A *soft anchor* is a reference level that the funding rule settles against but that is itself an estimate rather than a transaction price, so that deviations from it cannot be arbitraged (a pre-IPO (initial public offering) reference valuation is the leading case). The *observer gain* is the coefficient on the innovation in a filtering recursion, the weight the estimate places on a unit of surprise. We show in Section 8 that the funding intensity plays this role.

**Notation.** We write $E_{\cdot }^{Q}$ for $E^{Q}\left[\;\cdot |F_{t}\right]$ and reserve the unadorned E for expectations under P or for unconditional moments. The perpetual price is $f_{t}$ in the discrete-time construction of Section 2 and $F_{t}$ in the continuous-time framework used from Section 3 onward; the two are related by the usual limit and the distinction is only in the time index. The basis is written $\beta_{t}=F_{t}-S_{t}$ throughout. Differentials are set upright.

The reader interested only in the empirical episode may read Section 3, Section 8, Section 13, Section 14, and Section 15.

## 2. Shiller’s Construction

Shiller’s idea is to settle a contract every period against a flow index rather than against a transactable spot price. Let $f_{t}$ be the perpetual price at day *t*, let $D_{t}$ be the dividend or rent index for day *t*, and let $r_{t}$ be the one-period risk-free interest rate over [t,t+1]. We use the risk-free rate and not the return on a low-risk alternative: $r_{t}$ enters below as the discount rate in a present value under Q, and a low-risk asset whose return contains a risk premium would not serve that role. The daily cash settlement paid from short to long is

(1) $$\Delta \Sigma_{t}=D_{t}-r_{t}\;f_{t},$$

written as ΔΣ because it is the discrete counterpart of the cumulative settlement $\Sigma_{t}$ of Section 3. The long receives the index flow and pays financing on the contract price. The position is opened at zero cost, so, over [t,t+1], the long earns the capital gain plus the settlement:

$$\Pi_{t}=\left(f_{t+1}-f_{t}\right)+\Delta \Sigma_{t}=f_{t+1}-f_{t}+D_{t}-r_{t}f_{t}.$$

Imposing the no-arbitrage condition $E_{\Pi_{t}}^{Q}=0$ gives the pricing recursion

(2) $$f_{t}=\frac{E_{f_{t+1}}^{Q}+D_{t}}{1+r_{t}},$$

which iterates forward, under a transversality condition, to

(3) $$f_{t}=\sum_{k=0}^{\infty }\frac{E_{t}^{Q}\;\left[D_{t+k}\right]}{\prod_{j=0}^{k}\left(1+r_{t+j}\right)}.$$

The perpetual price equals the present value of the expected flow. With $r_{t}=r$ and E[D]=D constant, this collapses to the Gordon form f=D/r. The point is structural: the contract recovers a fundamental value using only an observable flow, so it prices assets that are never traded and whose spot is never seen. That is why Shiller aimed it at real estate, labor income, and price indices.

## 3. A Unified Perpetual: Present Value at the Funding Rate

Work in continuous time with a general settlement rule. Fix a risk-neutral measure Q and a filtration $\left(F_{t}\right)$. Consider a perpetual with price $F_{t}$ whose holder receives an instantaneous settlement

(4) $$d\Sigma_{t}=\left(a_{t}-b_{t}F_{t}\right)\;dt,$$

where $a_{t}$ and $b_{t}>0$ are adapted. We call $a_{t}$ the *benchmark flow* and $b_{t}$ the *settlement rate coefficient*: it is the coefficient on the price in the settlement rule, and its economic content depends on the design. In the Shiller specification of Corollary 1(i), it is a risk-free interest rate and discounts a dividend flow; in the crypto specification of Corollary 1(ii), it is the slope of the funding rule, and we then call it the *funding intensity* and write κ for it. We reserve the term funding intensity for that case, since calling an interest rate a funding intensity would obscure the very distinction the unification is meant to make. Both symbols carry a time index, $a_{t}$ and $b_{t}$, since neither is constant in general. The two existing designs are the two choices of $\left(a_{t},b_{t}\right)$ given below. Entry is costless, so the cumulative gain $G_{t}=F_{t}+\Sigma_{t}$ is a Q-martingale. Where a spot $S_{t}$ is defined, we write $\beta_{t}=F_{t}-S_{t}$ for the basis.

We collect the regularity conditions used throughout, so that the propositions below can be read without restating them.

**Assumption 1.**  The processes *a* and *b* are adapted, with $b_{t}>0$ and $\int_{0}^{\infty }b_{u}\;du=\infty$ almost surely, and $E_{t}^{Q}\int_{t}^{\infty }e^{-\int_{t}^{s}b_{u}du}\left|a_{s}\right|\;ds<\infty$. The transversality condition $\lim_{T\to \infty }E_{e^{-\int_{t}^{T}b_{u}du}F_{T}}^{Q}=0$ holds. Where a spot *S* appears, it is adapted and satisfies the integrability required by the displayed expectations. Martingale and optional sampling conditions are imposed separately in Corollaries 2 and 3, where they are used.

**Proposition 1** (Perpetual price as a present value at the funding rate)**.**  *Let $G_{t}=F_{t}+\Sigma_{t}$ be a Q-martingale with settlement $d\Sigma_{t}=\left(a_{t}-b_{t}F_{t}\right)\;dt$, $b_{t}>0$, and $\int_{0}^{\infty }b_{u}\;du=\infty$, and suppose the transversality condition $\lim_{T\to \infty }E_{t}^{Q}\left[e^{-\int_{t}^{T}b_{u}du}F_{T}\right]=0$ holds. Then,*

(5) $$F_{t}=E_{t}^{Q}\left[\int_{t}^{\infty }e^{-\int_{t}^{s}b_{u}\;du}\;a_{s}\;ds\right].$$

**Proof.**  The martingale property gives $E_{dF_{t}}^{Q}=-E_{d\Sigma_{t}}^{Q}=\left(b_{t}F_{t}-a_{t}\right)\;dt$. Write $\Lambda_{t}=\exp\left(-\int_{0}^{t}b_{u}\;du\right)$, so $d\left(\Lambda_{s}F_{s}\right)=\Lambda_{s}\left(dF_{s}-b_{s}F_{s}\;ds\right)$ and hence $E_{s}^{Q}\left[d\left(\Lambda_{s}F_{s}\right)\right]=\Lambda_{s}\left(E_{s}^{Q}\left[dF_{s}\right]-b_{s}F_{s}\;ds\right)=-\Lambda_{s}a_{s}\;ds$. Integrating from *t* to *T* and taking $E_{t}^{Q}$,

$$E_{\Lambda_{T}F_{T}}^{Q}-\Lambda_{t}F_{t}=-\;E_{t}^{Q}\int_{t}^{T}\Lambda_{s}a_{s}\;ds.$$

Divide by $\Lambda_{t}$, let T→∞, and apply transversality.    □

The perpetual price is the present value of the benchmark flow *a* discounted at the funding rate *b*. The funding rule fixes both the flow being averaged and the rate at which the average decays.

**Corollary 1** (The two designs)**.**  *(i) Shiller. With $a_{t}=D_{t}$ and $b_{t}=r_{t}$, $F_{t}=E_{t}^{Q}\int_{t}^{\infty }e^{-\int_{t}^{s}r_{u}du}D_{s}\;ds$, the present value of the dividend or rent flow at the risk-free rate, the continuous-time form of (3). (ii) Crypto basis peg. With observable spot $S_{t}$ and funding paid by the long $g_{t}=\kappa \;\beta_{t}$, the settlement received by the long is $-\kappa \beta_{t}\;dt$, so $a_{t}=\kappa S_{t}$ and $b_{t}=\kappa$, giving*

(6) $$F_{t}=\kappa \;E_{t}^{Q}\left[\int_{t}^{\infty }e^{-\kappa (s-t)}S_{s}\;ds\right],$$

*an exponentially weighted average of the expected future spot with horizon 1/κ.*

**Corollary 2** (The peg and its fast funding limit)**.**  *If S is a uniformly integrable Q-martingale, then $F_{t}=S_{t}$ for every κ>0. More generally, if $s\mapsto E_{S_{s}}^{Q}$ is right continuous at s=t and the family $\left\{S_{s}:s\in \left[t,t+\epsilon \right]\right\}$ is conditionally uniformly integrable for some ϵ>0, then $F_{t}\to S_{t}$ as κ→∞.*

**Proof.**  The kernel $\kappa e^{-\kappa (s-t)}$ is the density of t+Exp(κ), of unit mass. If $E_{S_{s}}^{Q}=S_{t}$, then (6) averages a constant and returns $S_{t}$. As κ→∞, the density converges weakly to the point mass at *t*, and right continuity of $s\mapsto E_{S_{s}}^{Q}$ gives $F_{t}\to S_{t}$.    □

Shiller’s design is self-anchoring: it settles on a real flow and needs no external price. The crypto design is basis-anchored: it needs an observable spot, and the peg is exact only in the fast funding limit or when the spot is a Q-martingale. This is the precise sense in which the two are the same instrument with two benchmarks.

## 4. The Perpetual as a Random Time Change

Proposition 1 has a probabilistic reading that is useful for the stochastic volatility analysis that follows. Take the peg form $a_{s}=b_{s}S_{s}$ so that a/b=S. This is the crypto contract of Corollary 1(ii) with a possibly time-varying intensity. Then, (5) becomes

(7) $$F_{t}=E_{t}^{Q}\left[\int_{t}^{\infty }b_{s}\;e^{-\int_{t}^{s}b_{u}\;du}\;S_{s}\;ds\right].$$

The kernel $b_{s}\exp\left(-\int_{t}^{s}b_{u}\;du\right)$ is the density in *s* of the first event after *t* of a Cox process with intensity *b*. Writing $\tau =\inf\{s\geq t:\int_{t}^{s}b_{u}\;du\geq E\}$ with E∼Exp(1) independent of F, the price is an expected spot at a random time.

**Proposition 2** (Random time change)**.**  *For the peg form, $F_{t}=E_{S_{\tau }}^{Q}$, the expected spot at the first event of a clock whose intensity is the funding rate.*

**Proof.**  Condition on the path. The law of τ given F has density $b_{s}e^{-\int_{t}^{s}b_{u}du}$ on [t,∞), so

$$E_{S_{\tau }}^{Q}=E_{t}^{Q}\int_{t}^{\infty }b_{s}e^{-\int_{t}^{s}b_{u}du}S_{s}\;ds,$$

which is the right side of (7).    □

**Figure 1:**
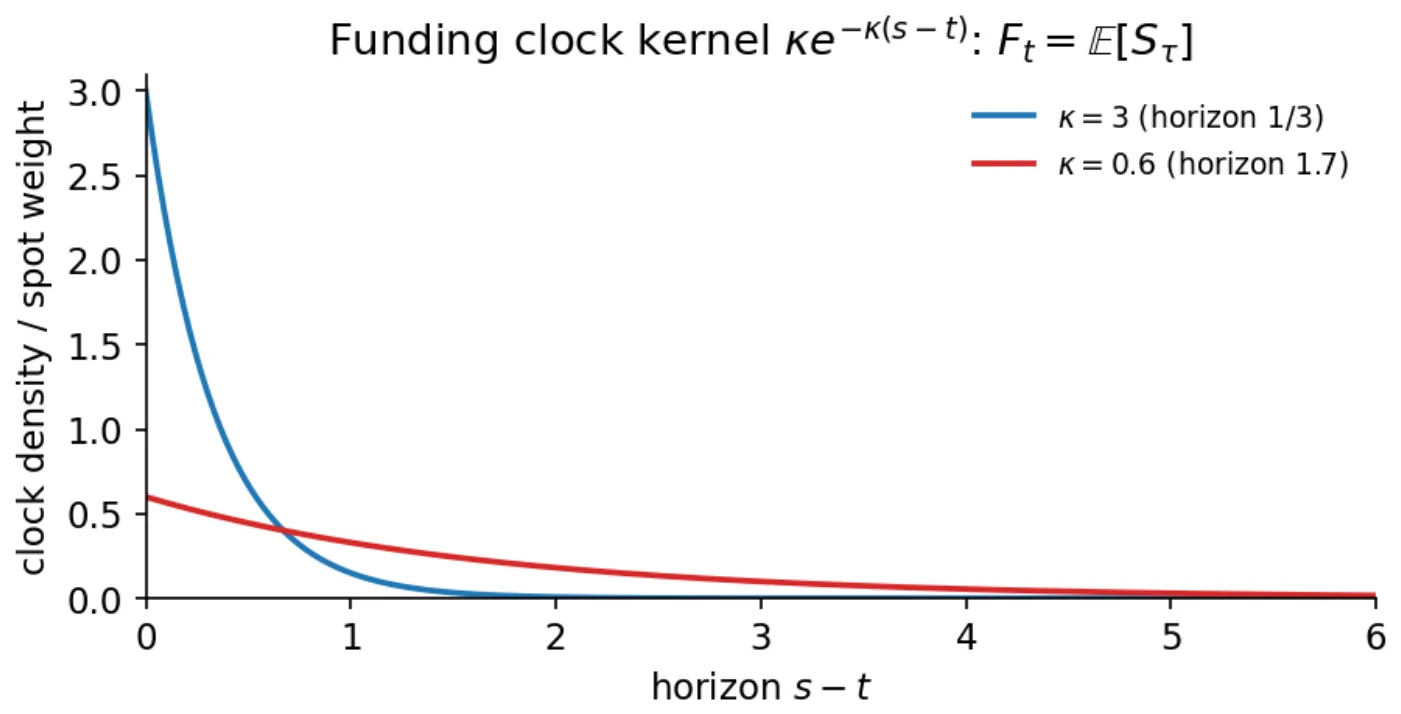
The perpetual averages the future spot against the funding clock kernel $\kappa e^{-\kappa (s-t)}$. A high funding intensity κ concentrates the weight near the present and tightens the peg; a low intensity samples further out. The price is the expected spot at the first event of this clock.

With constant b=κ, the clock is Poisson and τ=t+Exp(κ), recovering the exponentially weighted average of Corollary 1(ii). The peg is now transparent. If *S* is a Q-martingale and the clock is independent of *S*, optional sampling gives $E_{S_{\tau }}^{Q}=S_{t}$, so $F_{t}=S_{t}$ exactly, for any intensity. Independence is sufficient, not necessary. Corollary 3 enlarges the filtration by the exponential mark and shows that the peg remains exact whenever *S* stays a uniformly integrable martingale there, even when the clock and spot share a volatility state. Only the carry survives in the basis. Figure 1 shows this weighting.

This is a random time change: the funding rule samples the spot at the first event of the clock τ. A single Cox first-event time is not itself an increasing Lévy subordinator. With constant intensity, it generates the exponential first-event family, and varying the intensity generates the corresponding Bochner-style family of clocks.

## 5. A No-Arbitrage Band Under Capped Funding

Real contracts cap funding, $|g_{t}|\leq \bar{g}$, and the basis trade that would enforce the peg is not free. Suppose an arbitrageur who is long spot and short perpetual when $F_{t}>S_{t}$ pays a carry rate χ>0 for margin, collateral and borrow, and collects funding $g_{t}=\min\left\{\kappa \beta_{t},\;\bar{g}\right\}$.

**Proposition 3** (Funding enforceable band)**.**  *In the uncapped regime, the basis is pinned at $\beta_{t}=\chi /\kappa$. Funding can discipline the basis only when $\chi \leq \bar{g}$ and while $\kappa \;|\beta_{t}|\leq \bar{g}$, which is inside the band*

(8) $$|\beta_{t}|\leq \frac{\bar{g}}{\kappa }.$$

*Outside this band, funding saturates at $\bar{g}$ and the only remaining discipline is a convergence trade, which requires a view on S.*

**Proof.**  The basis trade earns $g_{t}-\chi$ per unit time. While uncapped, $g_{t}=\kappa \beta_{t}$, and entry continues until $g_{t}=\chi$, giving $\beta_{t}=\chi /\kappa$, which lies inside the band only if $\chi \leq \bar{g}$. Funding rises with the basis only until $\kappa \beta_{t}=\bar{g}$. Beyond that point, $g_{t}\equiv \bar{g}$ regardless of the basis, so a larger basis cannot be removed by collecting funding, and closing it requires betting that *F* will converge to *S*.    □

The band $\bar{g}/\kappa$ is tight when κ is large and the spot is hard. For a private firm, there is no hard *S*, so κ is keyed to a reference estimate and the band is centered on a soft number. Ackerer, Hugonnier and Jermann [5] give sharp deviation results in the frictional case; Gromb and Vayanos [10] provide the broader limits-of-arbitrage interpretation.

## 6. Equivalence with a Hard Spot

When a genuine tradable spot exists, the choice of benchmark does not matter: both designs return the spot.

**Proposition 4** (Equivalence with a hard spot)**.**  *Let $S_{t}$ be a tradable spot paying dividend flow $D_{t}=q_{t}S_{t}$, so that, under Q, $E_{dS_{t}}^{Q}=\left(r_{t}-q_{t}\right)S_{t}\;dt$. Then,*

*(i)*  *The Shiller dividend-settled perpetual satisfies $F_{t}=S_{t}$;*
*(ii)*  *A basis-funded perpetual satisfies $F_{t}=S_{t}$ if and only if its settlement pair obeys*
(9) $$a_{t}=\left(b_{t}-r_{t}+q_{t}\right)\;S_{t}.$$
*In particular, the interest-adjusted funding rule $g_{t}=\kappa \left(F_{t}-S_{t}\right)+\left(r_{t}-q_{t}\right)F_{t}$, for which $a_{t}=\kappa S_{t}$ and $b_{t}=\kappa +r_{t}-q_{t}$, returns $F_{t}=S_{t}$ for every κ>0.*

**Proof.**  (i) The Shiller perpetual has $E_{dF_{t}}^{Q}=\left(r_{t}F_{t}-D_{t}\right)\;dt$ from Section 2. With $\beta_{t}=F_{t}-S_{t}$, the two drifts subtract to $E_{d\beta_{t}}^{Q}=r_{t}\beta_{t}\;dt$, so $\Lambda_{t}^{r}\beta_{t}$ with $\Lambda_{t}^{r}=e^{-\int_{0}^{t}r_{u}du}$ is a Q-martingale. Under the transversality condition $\lim_{T\to \infty }E_{\Lambda_{T}^{r}\beta_{T}}^{Q}=0$, this forces $\beta_{t}=0$.

(ii) By the martingale property of *G*, $E_{dF_{t}}^{Q}=\left(b_{t}F_{t}-a_{t}\right)\;dt$. Substituting the candidate $F_{t}=S_{t}$ and matching against $E_{dS_{t}}^{Q}=\left(r_{t}-q_{t}\right)S_{t}\;dt$ gives $b_{t}S_{t}-a_{t}=\left(r_{t}-q_{t}\right)S_{t}$, which is (9). The argument reverses, and uniqueness follows as in (i). For the stated rule, the long receives $-g_{t}\;dt$, so $a_{t}=\kappa S_{t}$ and $b_{t}=\kappa +r_{t}-q_{t}$, and (9) holds identically. Equivalently, by Proposition 1 with deterministic rates, $F_{t}=\kappa S_{t}\int_{0}^{\infty }e^{-(\kappa +r-q)u}e^{(r-q)u}du=S_{t}$.    □

**Remark 1.**  *Condition (9) is what makes the funding rule interest-adjusted, and it is easy to get wrong. A rule that credits the carry to the benchmark flow rather than to the settlement rate coefficient, taking $a_{t}=\left(r_{t}-q_{t}+\kappa \right)S_{t}$ with $b_{t}=\kappa +r_{t}$, delivers $F_{t}=S_{t}$ only in the knife-edge case $r_{t}=2q_{t}$; in general, for constant coefficients, Proposition 1 gives*

$$F_{0}=\frac{\kappa +r-q}{\kappa +q}S_{0},\;\beta_{0}=S_{0}\frac{r-2q}{\kappa +q}.$$

*Section 13 verifies the corrected rule numerically and reports the agreement of the misspecified rule with this exact basis.*

So the difference between the designs is entirely about the case where there is no hard spot. There, Shiller settles on a flow that does exist, while the crypto contract must invent a reference.

## 7. Stochastic Volatility Enters Only Through Carry

Proposition 2 writes the peg as $F_{t}=E_{S_{\tau }}^{Q}$, the expected spot at the funding clock. This pins down the role of volatility exactly.

**Proposition 5** (Exact basis)**.**  *Let $dS_{t}=c_{t}S_{t}\;dt+S_{t}\sqrt{v_{t}}\;dW_{t}$ under Q with adapted funding intensity $b_{t}>0$. For the peg form,*

(10) $$\beta_{t}=E_{t}^{Q}\left[\int_{t}^{\infty }e^{-\int_{t}^{s}b_{u}\;du}\;c_{s}S_{s}\;ds\right].$$

**Proof.**  Let $\Lambda_{s}=e^{-\int_{t}^{s}b_{u}du}$, which is continuous and of finite variation, so $b_{s}\Lambda_{s}\;ds=-d\Lambda_{s}$ and $d\left(\Lambda_{s}S_{s}\right)=\Lambda_{s}\;dS_{s}+S_{s}\;d\Lambda_{s}$. By (7) and integration by parts, with $\Lambda_{t}=1$ and $\Lambda_{\infty }=0$,

$$F_{t}=E_{t}^{Q}\int_{t}^{\infty }S_{s}\left(-d\Lambda_{s}\right)=E_{t}^{Q}\left[S_{t}+\int_{t}^{\infty }\Lambda_{s}\;dS_{s}\right].$$

The integrand Λ is bounded and predictable, so the stochastic integral against the local martingale part of *S* has a zero mean, leaving $E_{t}^{Q}\int_{t}^{\infty }\Lambda_{s}c_{s}S_{s}\;ds$.    □

**Corollary 3** (Volatility irrelevance)**.**  *If c≡0 and S is a uniformly integrable Q-martingale on the filtration enlarged by the exponential mark, then $F_{t}=S_{t}$ exactly for every funding intensity, including one driven by the same state as S and under any leverage d〈W,v〉≠0. Stochastic volatility alone does not move the basis.*

The corollary is optional sampling: in the filtration enlarged by the exponential mark, τ is a stopping time and *S* stays a martingale, so $E_{S_{\tau }}^{Q}=S_{t}$. Volatility reaches the basis only through the carry *c*. If a volatility risk premium makes $c_{t}=c\left(v_{t}\right)$, then (10) is the funding discounted expected carry, whose sign is the sign of the priced carry and whose size is a covariance between the funding discount and that carry. This is the perpetual analogue of the futures-minus-forward adjustment of Cox, Ingersoll and Ross [11]: the gap is the covariance between the discount, here funding, and the asset’s drift, and it vanishes when the drift is zero or the funding is deterministic. To trade the volatility itself, one writes a perpetual on realized variance (Section 16). Its benchmark flow is *v*, not a martingale spot, so Corollary 3 does not apply and it carries the exposure.

Corollary 3 applies under a Q-martingale spot, a funding clock that may depend on the same state, and the integrability conditions of Assumption 1. Under those conditions, volatility reaches the basis only through the carry *c*. The corollary does not imply that empirical bases are independent of realized volatility. Volatility moves margin requirements, liquidation risk, and the cost of the basis trade, so it moves χ in Proposition 3 and hence the width of the enforceable band. It moves the risk-bearing capacity $\gamma_{U}$ and hence the segmentation basis of Proposition 6, and, in a stressed market, it moves the depth on both sides at once. The corollary isolates one channel and closes it. The other channels operate through the frictions, which is where the model puts them, and separating the two is exactly what the estimation of Section 11 is for.

## 8. Price Discovery as Filtering

The pre-IPO problem is that the fundamental is latent. The first object is the *latent fundamental* $x_{t}$, the log value of the firm, which is never observed. The second is the *observation process* $y_{t}$, a cumulative noisy signal generated by reference marks, funding round prints and order flow [12]. $y_{t}$ is not itself a valuation, and only its increments carry information. The third is the *filtered estimate* ${\hat{x}}_{t}=E\left[x_{t}|F_{t}^{y}\right]$, the market’s conditional mean given everything observed by *t*, which is the object the traded price tracks. Confusing the second and third is the natural error, because both are observable; the distinction is that $y_{t}$ accumulates evidence while ${\hat{x}}_{t}$ summarizes it.

Let the latent fundamental follow

(11) $$dx_{t}=\mu \;dt+\sigma \;dW_{t},$$

and let the observation process satisfy

(12) $$dy_{t}=x_{t}\;dt+\eta \;dB_{t},$$

with W,B being independent and η the reference noise. Write ${\hat{x}}_{t}=E\left[x_{t}|F_{t}^{y}\right]$ for the filtered estimate and $P_{t}=\mathrm{Var}\left(x_{t}|F_{t}^{y}\right)$ for its posterior variance. Linear Gaussian filtering gives the correction form [13]

(13) $$d{\hat{x}}_{t}=\mu \;dt+\frac{P_{t}}{\eta^{2}}\;\left(dy_{t}-{\hat{x}}_{t}\;dt\right),\;{\dot{P}}_{t}=\sigma^{2}-\frac{P_{t}^{2}}{\eta^{2}},$$

with steady-state $P_{\infty }=\sigma \eta$ and steady-state gain $K_{\infty }=P_{\infty }/\eta^{2}=\sigma /\eta$. The innovation $dy_{t}-{\hat{x}}_{t}\;dt$ is the part of the reference that was not already anticipated. The units match after writing the funding correction in logs: if $f_{t}=\log F_{t}$ and $s_{t}=\log S_{t}$, then the local correction is $-\kappa (f_{t}-s_{t})\;dt$. Thus, $s_{t}$ plays the role of the observation and κ the role of the gain $K_{t}$. In levels, $f_{t}-s_{t}\approx \beta_{t}/S_{t}$, which supplies the required price scaling. We use *observer gain* for this coefficient throughout: it is the weight the price places on a unit of surprise, large when the signal is precise relative to the motion of the fundamental and small when it is not. In these terms, the funding rule is a feedback observer and the peg is its fixed point.

This reading makes the pre-IPO limitation precise. Before listing, there is no observation equation tied to an exogenous transaction price: the reference $y_{t}$ is itself an estimate, so η is large, the steady-state variance $P_{\infty }=\sigma \eta$ is large, and the gain is small. The estimate is a wide posterior that order flow can move, and no external term pulls it back. At the Nasdaq open, a hard observation arrives with η→0, the gain diverges, the posterior collapses onto the print, and the perpetual rebases. ‘Arbitrage closed’ is precisely the statement that an exogenous observation with η→0 is present. Before that, discovery is real, because order flow does update ${\hat{x}}_{t}$, but it is not closed, because nothing observes $x_{t}$ directly.

**Figure 2:**
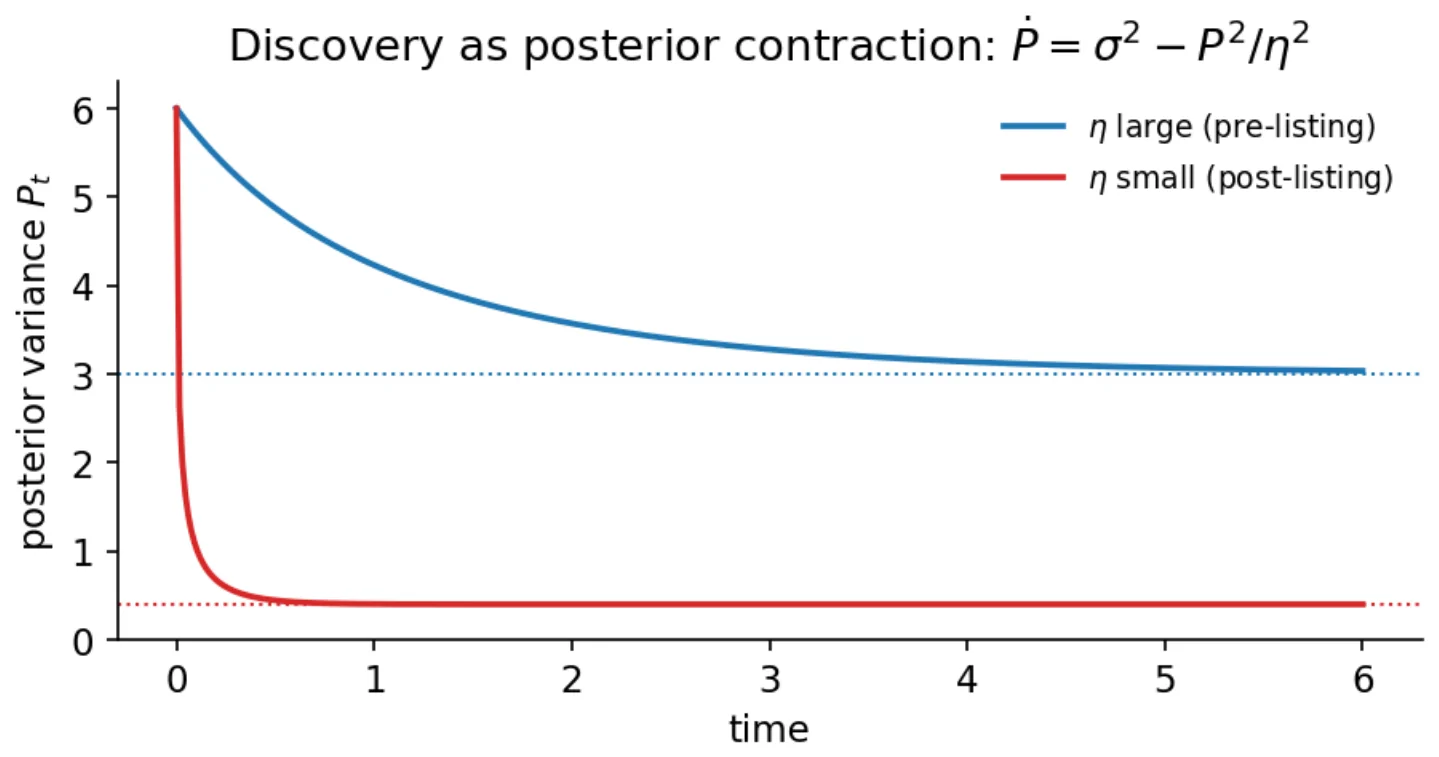
The posterior variance $P_{t}$ solving $\dot{P}=\sigma^{2}-P^{2}/\eta^{2}$ contracts to its steady-state $P_{\infty }=\sigma \eta$. A large observation noise η, the pre-listing regime in which the only signal is a soft reference, leaves a wide posterior; a small η, the hard observation supplied at the Nasdaq open, collapses it. Discovery is the contraction of this variance.

A mean-reverting fundamental sharpens the picture. If, instead, $dx_{t}=\phi \left(\bar{x}-x_{t}\right)\;dt+\sigma \;dW_{t}$, the steady-state posterior variance solves $0=\sigma^{2}-2\phi P_{\infty }-P_{\infty }^{2}/\eta^{2}$, giving $P_{\infty }=\eta^{2}\left(\sqrt{\phi^{2}+\sigma^{2}/\eta^{2}}-\phi \right)$, which is increasing in the observation noise η and in the innovation variance $\sigma^{2}$ and decreasing in the mean reversion speed ϕ. A firm with a stable, well-anchored fundamental is easy to discover even through a noisy reference, while a firm whose value is itself a moving target stays uncertain until a hard observation arrives. The steady-state gain $K_{\infty }=P_{\infty }/\eta^{2}$ is the funding intensity a market would have to run in order to track the fundamental at the filter’s rate, which links the choice of κ to the primitives ϕ, σ, and η. Figure 2 shows this contraction.

## 9. Bayesian Martingale Structure

Three martingale facts organize the discovery story. Each is standard, and, together, they say what the perpetual is doing.

**The price is a Doob martingale of the latent value.** Let $x_{T}\in L^{1}\left(F_{\infty }\right)$ be the log fundamental of Section 8 evaluated at the listing date *T*, the value the listing reveals, and write $p_{t}=E\left[x_{T}|F_{t}\right]$ for the price as a predictor of it. This is a different conditional mean from the filtered estimate ${\hat{x}}_{t}=E\left[x_{t}|F_{t}^{y}\right]$: the filter estimates where the fundamental is now, the price predicts where it will be found at listing, and the two coincide when the fundamental is a martingale, that is, when μ=0 in (11). Then, $\left(p_{t}\right)$ is a uniformly integrable martingale, and, by the martingale convergence theorem, $p_{t}\to x_{T}$ almost surely and is in $L^{1}$ as $F_{t}↑F_{\infty }$. The conditional variance $\mathrm{Var}(x_{T}|F_{t})$ decreases, and its drop is the information the market has acquired. The listing is the time at which a hard observation makes that drop discontinuous. This is the filtering of Section 8 stated as a convergence theorem [14]: price discovery is the convergence of a Doob martingale, and the funding rule is the mechanism that moves $p_{t}$ on each innovation. This martingale is in logs. The corresponding level predictor is $L_{t}=E\left[e^{x_{T}}|F_{t}\right]$, not $e^{p_{t}}$; their difference is the Jensen correction generated by conditional uncertainty.

**Cumulative settlement is a martingale difference.** Under the pricing measure, the per-period settlement satisfies $E_{s_{t}+\Delta F_{t}}^{Q}=0$ by construction, so the cumulative gain $G_{t}=F_{t}+\Sigma_{t}$ is a Q-martingale by Proposition 1. The perpetual is free of arbitrage if and only if such an equivalent martingale measure exists, that is, the fundamental theorem of asset pricing localized to this contract [8,15]. The settlement stream is, up to sign, realized minus predicted, so a correctly priced perpetual is a tradeable prequential score of the predictive sequence: it pays when the prediction is wrong and nets to a martingale when the predictions are calibrated.

**Funding is a stochastic approximation.** Discretize and let order flow move the price toward the target $m_{k}=E\left[x_{T}|F_{k}\right]$ in response to the funding signal $g_{k}=\kappa \beta_{k}$,

(14) $$F_{k+1}-F_{k}=-\alpha_{k}\;g_{k}+\xi_{k+1},\;E\left[\xi_{k+1}|F_{k}\right]=0.$$

This is a Robbins and Monro [16] recursion with a fixed-point posterior mean. With a decreasing gain satisfying $\sum_{k}\alpha_{k}=\infty$ and $\sum_{k}\alpha_{k}^{2}<\infty$, it converges to $m_{k}$ when the target is fixed; with the constant gain of a live contract, it tracks a moving target, which is what a nonstationary fundamental requires. The product $\alpha_{k}\kappa$ is the effective learning rate, so the funding intensity sets the speed at which the market moves toward its own posterior, and the peg is the fixed point. This is the same correction as the observer gain of Section 8, now read as stochastic optimization toward $E[x_{T}|F_{t}]$.

## 10. A Segmented Market Equilibrium for the Basis

The premium decomposition of Section 15 treats segmentation as a residual. Here, it is given structure. The perpetual is in zero-net supply, so every long is matched by a short. Constrained agents, such as retail or non-US investors, can hold only the perpetual. Let $n_{c}\geq 0$ denote their aggregate desired long position at zero segmentation premium. The unconstrained side can hold the IPO allocation or bear the listing risk and takes the short side.

The equilibrium object must be distinguished from the observed basis. Let ${\bar{V}}_{t}=E_{t}\left[\tilde{V}\right]$ be the expected listing value, define the segmentation premium $\psi_{t}=F_{t}-{\bar{V}}_{t}$, and define the reference gap $\omega_{t}={\bar{V}}_{t}-S_{t}$. Then,

$$\beta_{t}=F_{t}-S_{t}=\psi_{t}+\omega_{t}.$$

Before listing, the soft reference $S_{t}$ need not equal ${\bar{V}}_{t}$.

**Proposition 6** (Segmentation basis)**.**  *Let the listing value be $\tilde{V}$ with $E\tilde{V}=\bar{V}$ and $\mathrm{Var}\;\tilde{V}=\sigma_{V}^{2}$, and let $\gamma_{U}$ be the aggregate risk tolerance of the unconstrained side. In the uncapped zero-net-supply equilibrium,*

(15) $$\psi_{t}=\lambda n_{c},\;\lambda =\frac{\sigma_{V}^{2}}{\gamma_{U}},\;\beta_{t}=\lambda n_{c}+\omega_{t}.$$

*This equilibrium is inside the funding-enforceable band only when $|\lambda n_{c}+\omega_{t}|\leq \bar{g}/\kappa$. If the reference is unbiased, $S_{t}={\bar{V}}_{t}$ and $\omega_{t}=0$, the observed basis equals the segmentation premium.*

**Proof.**  A short position of *q* units earns $q(F-\tilde{V})$ at listing. The unconstrained side therefore chooses *q* to maximize $q\left(F-\bar{V}\right)-q^{2}\sigma_{V}^{2}/\left(2\gamma_{U}\right)$, giving short supply $q^{⋆}=\gamma_{U}\psi /\sigma_{V}^{2}$. Clearing against constrained demand, $q^{⋆}=n_{c}$, which gives $\psi =\sigma_{V}^{2}n_{c}/\gamma_{U}$. Adding the reference gap $\omega =\bar{V}-S$ gives the observed basis in (15). The final condition is Proposition 3 applied to that observed basis.    □

The comparative statics are the economics of the section. As $\gamma_{U}\to \infty$, the segmentation premium vanishes. The observed basis also vanishes when the reference becomes the hard spot, so $\omega_{t}=0$. As risk-bearing capacity falls, through scarce borrow or capital limits, $\psi_{t}$ widens in proportion to constrained demand. For a private firm, there is no borrow and the IPO allocation is scarce, so $\gamma_{U}$ is small and $n_{c}$ is large. The observed basis may also contain the separate reference gap $\omega_{t}$. The segmentation premium is structural, a price of access and risk bearing rather than a forecast error, and it should decay as listing and the staggered lock-up releases restore risk-bearing capacity. If the implied basis lies outside the funding band, Proposition 3 alone does not pin the price.

## 11. Estimation by Generative Bayesian Computation

The model that has accumulated, a latent fundamental with stochastic volatility observed through a capped funding mechanism, is a nonlinear non-Gaussian state-space model, and the Gaussian filter of Section 8 is only its linear special case. Inference on the latent path and the parameters $\Theta =(\Theta_{s},c,\iota ,\bar{g})$, with state-space block $\Theta_{s}=\left(\mu ,\sigma ,\eta ,\kappa \right)$, from the observed series of price, funding, and volume is therefore likelihood free in practice. Generative Bayesian computation [9] replaces the likelihood with simulation and a learned posterior map:

1. Draw $\Theta^{\left(i\right)}$ from the prior, simulate a path of price, funding and volume, and reduce each to a vector of summary statistics $z^{\left(i\right)}$;
2. Train a conditional density or quantile estimator z↦Θ on the pairs $\left(z^{\left(i\right)},\Theta^{\left(i\right)}\right)$;
3. Evaluate the estimator at the observed $z^{⋆}$ to draw from the posterior $\Theta |z^{⋆}$;
4. Propagate to the filtered fundamental ${\hat{x}}_{t}$ and the basis decomposition of Section 15 with posterior uncertainty.

The informative summaries are the mean and persistence of the funding rate, which identify κ and the band, the realized volatility and the level of the funding carry, which identify σ and *c* of Section 7, and the basis level and its mean reversion, which identify the segmentation term. The output is a posterior over the carry basis and the premium components rather than point estimates.

This is the procedure that would identify the decomposition (17): A prior on $\left(\gamma_{U},n_{c},\sigma_{V}\right)$ induces a prior on the segmentation term $\zeta_{t}$ through Proposition 6, and a prior on the carry $c_{t}$ induces one on the risk premium $\rho_{t}$ through Proposition 5. The bubble term is then the posterior of the remainder, rather than an algebraic residual, and comes with a credible interval. The 73 complete-day Hyperliquid sample in Section 14 is short relative to funding rate persistence, so a posterior for the empirical decomposition would be prior-dominated. Section 13.2 instead evaluates parameter recovery on simulated tapes where the truth is known. Estimation of the decomposition requires a longer panel of perpetual contracts.

## 12. Discrete Funding Mechanics

The continuous settlement (7) is an idealization of a discrete rule. A live contract marks to a mark price $M_{t}$ and references an index price $I_{t}$, and, at funding times spaced by *h*, the long pays the short

(16) $$g_{t}=hN_{t}\left[\iota +\mathrm{clip}\left(\kappa \frac{M_{t}-I_{t}}{I_{t}},-\bar{r},\bar{r}\right)\right],$$

where $N_{t}$ is notional, ι is the fixed-rate component, and $\bar{r}$ is the cap on the premium-rate component. Hyperliquid settles hourly, while Binance USD-M settles at eight-hour intervals in this sample. If a venue quotes a per-interval unit coefficient on the relative premium, the continuous slope with respect to the absolute basis is $1/(hI_{t})$ per unit notional.

The mapping to Proposition 1 is immediate outside the cap. For one unit of underlying, set $N_{t}=I_{t}$ and $M_{t}=F_{t}$. Since the long receives $-g_{t}$,

$$-\frac{g_{t}}{h}=\left(\kappa -\iota \right)I_{t}-\kappa F_{t},\;a_{t}=\left(\kappa -\iota \right)I_{t},\;b_{t}=\kappa .$$

Thus, κ is the continuous funding slope, and the premium flow cap in the units of Proposition 3 is $\bar{g}=I_{t}\bar{r}$. The symbol ι is distinct from the carry $c_{t}$ of Section 7, which is a property of the underlying rather than of the contract. Between funding times, the contract is disciplined only by the mark mechanism and liquidations, so the band of Proposition 3 is a statement about averages across funding intervals rather than an instantaneous bound. For a private underlying, the index $I_{t}$ is a reference valuation rather than a transaction price, that is, the soft anchor analyzed in Section 1, Section 8 and Section 12.

## 13. Numerical Verification and Calibration

The SpaceX episode supplies a single realized path. Simulation therefore tests the pricing results against known closed forms and measures parameter recovery for the estimator of Section 11. An illustrative calibration then compares the implied valuation interval with the observed prices.

### 13.1. Pricing with an Observable Spot

Take a Heston spot under Q,

$$dS_{t}=c\;S_{t}\;dt+S_{t}\sqrt{v_{t}}\;dW_{t},\;dv_{t}=\kappa_{v}\left(\bar{v}-v_{t}\right)\;dt+\xi \sqrt{v_{t}}\;dZ_{t},\;d{\langle W,Z\rangle }_{t}=\rho_{S}\;dt,$$

with $S_{0}=100$, $v_{0}=\bar{v}=0.16$ and $\kappa_{v}=2$, and a settlement rate coefficient $b_{t}=\kappa_{0}+\kappa_{1}v_{t}$ that is allowed to depend on the same volatility state as the spot. The perpetual is priced by Proposition 1 using the within-step weight decomposition $\int_{0}^{\infty }b_{s}e^{-A_{s}}S_{s}\;ds=\sum_{k}S_{k}\left(e^{-A_{k}}-e^{-A_{k+1}}\right)$, where $A_{s}=\int_{0}^{s}b_{u}\;du$, so the discount weights sum to one by construction and no discretization bias enters through them. Here, $\kappa_{v}$ is the variance mean reversion speed, while $\kappa_{0}$ and $\kappa_{1}$ parameterize the funding clock. All are distinct from the contract intensity κ. Each table and figure below uses $2\times 10^{5}$ antithetic paths. Variance is advanced by a full-truncation Euler scheme, which accommodates the two ξ=1 configurations that violate the Feller condition.

Table 1 tests Corollary 3. When the spot is a Q-martingale, the basis is statistically indistinguishable from zero in every configuration: across the six clocks, the *t* statistic for $F_{0}=S_{0}$ ranges from −0.19 to +0.05 against Monte Carlo standard errors between 0.0087 and 0.0306, so no configuration rejects $F_{0}=S_{0}$ at any conventional level. This holds, including a funding clock driven by the volatility state itself ($\kappa_{1}=50$) and strong leverage of either sign. This is the content of the corollary: neither the level of volatility nor its volatility nor its correlation with the spot moves the peg.

**Figure 3:**
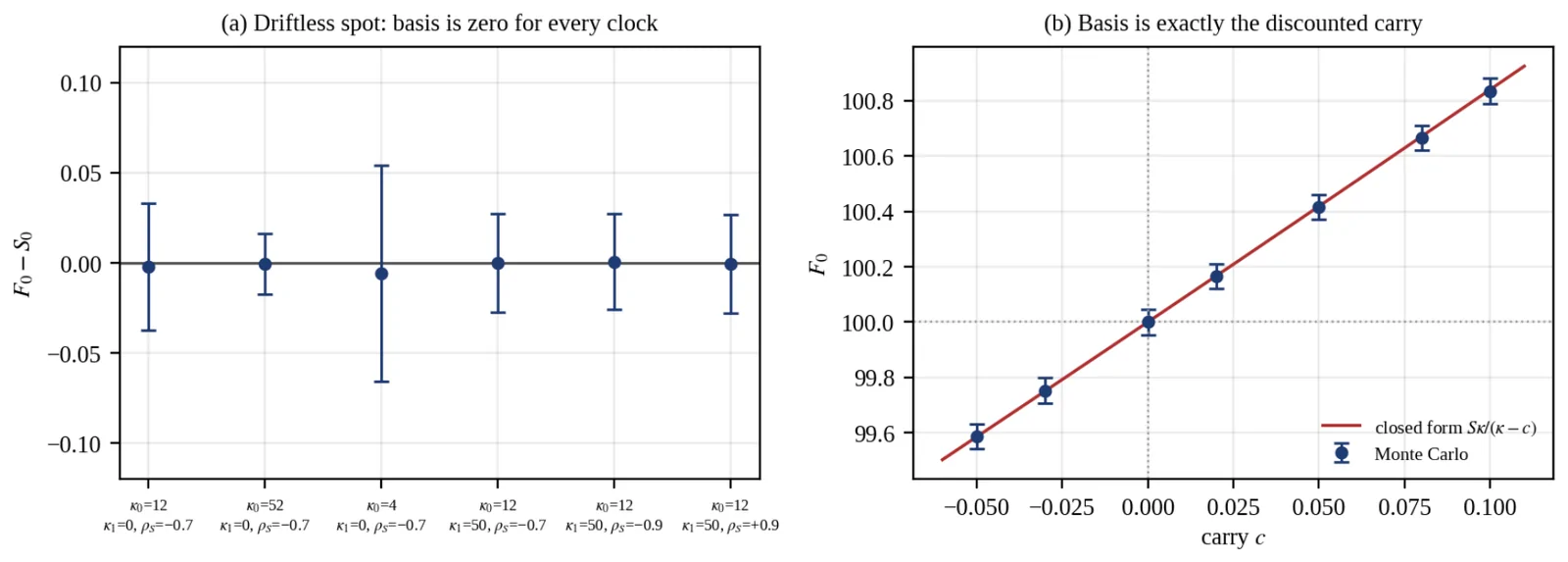
**Left**: the basis for a driftless spot, with 95 percent Monte Carlo intervals, across funding clocks that vary in intensity, in state dependence ($\kappa_{1}$) and in leverage ($\rho_{S}$). **Right**: the perpetual price against the carry *c*, with the closed form $S_{0}\kappa /\left(\kappa -c\right)$ of Proposition 5 overlaid. Volatility enters the basis only through the carry.

Table 2 tests Proposition 5. With a constant carry and constant intensity, the exact basis integrates to $F_{0}=S_{0}\kappa /\left(\kappa -c\right)$, and the simulation matches this to a relative error below $10^{-4}$ throughout, within the Monte Carlo standard errors of 0.0087 to 0.0182 reported in the table, for the carry of either sign. Figure 3 plots both results.

Proposition 4(ii) is evaluated with an observable spot. Take r=0.05, q=0.02, and κ=12, so that the spot drifts at r−q under Q. The interest-adjusted rule $a_{t}=\kappa S_{t}$, $b_{t}=\kappa +r-q$ returns $F_{0}=99.9666$ against a truncated closed form of 99.9665 with a standard error of 0.0201, a *t* statistic of 0.01. The rule discussed in the remark following Proposition 4, which credits the carry to the benchmark flow rather than to the settlement rate coefficient, returns 100.0502 on the same paths, a Monte Carlo basis of 8.4 basis points, consistent with the exact 8.3 basis points from the preceding remark. The framework therefore reproduces an observable spot, and does so only under (9).

### 13.2. Recovering the Parameters

Parameter recovery is evaluated under the model of Section 8 and Section 9: a latent log fundamental with drift μ and volatility σ, a reference signal with noise η, a market that runs the Kalman recursion on that signal, and a traded log price driven toward the resulting index by the funding rule at intensity κ, with an independent price noise $s_{F}$ treated as a nuisance parameter. This experiment fixes the contract parameters $\left(c,\iota ,\bar{g}\right)$ and estimates only the state-space block $\Theta_{s}=\left(\mu ,\sigma ,\eta ,\kappa \right)$; $s_{F}$ is drawn as an unreported nuisance. The econometrician observes only what a venue tape provides, namely, the price path and the funding path; the index is latent, so κ cannot be read off a regression of funding on an observed basis and must be identified from the joint dynamics. Each simulated record is one year of daily data, which is the length a single listed contract would supply.

We draw $8\times 10^{4}$ parameter vectors from a diffuse prior, simulate, reduce each path to eighteen summaries, train quantile regressions $z\mapsto \Theta_{s}$ at the 5th, 50th, and 95th percentiles, and evaluate on $3\times 10^{3}$ held-out draws. The eighteen summaries are the mean, standard deviation, mean absolute value, and autocorrelations at lags 1, 2, 5, and 10 of price increments; the mean, standard deviation, and autocorrelations at lags 1, 2, and 5 of funding; price–funding cross-correlations at lags 0 and 1; price–increment variance ratios at horizons 5, 10, and 25 days; and the log ratio of funding to price increment standard deviations.

**Figure 4:**
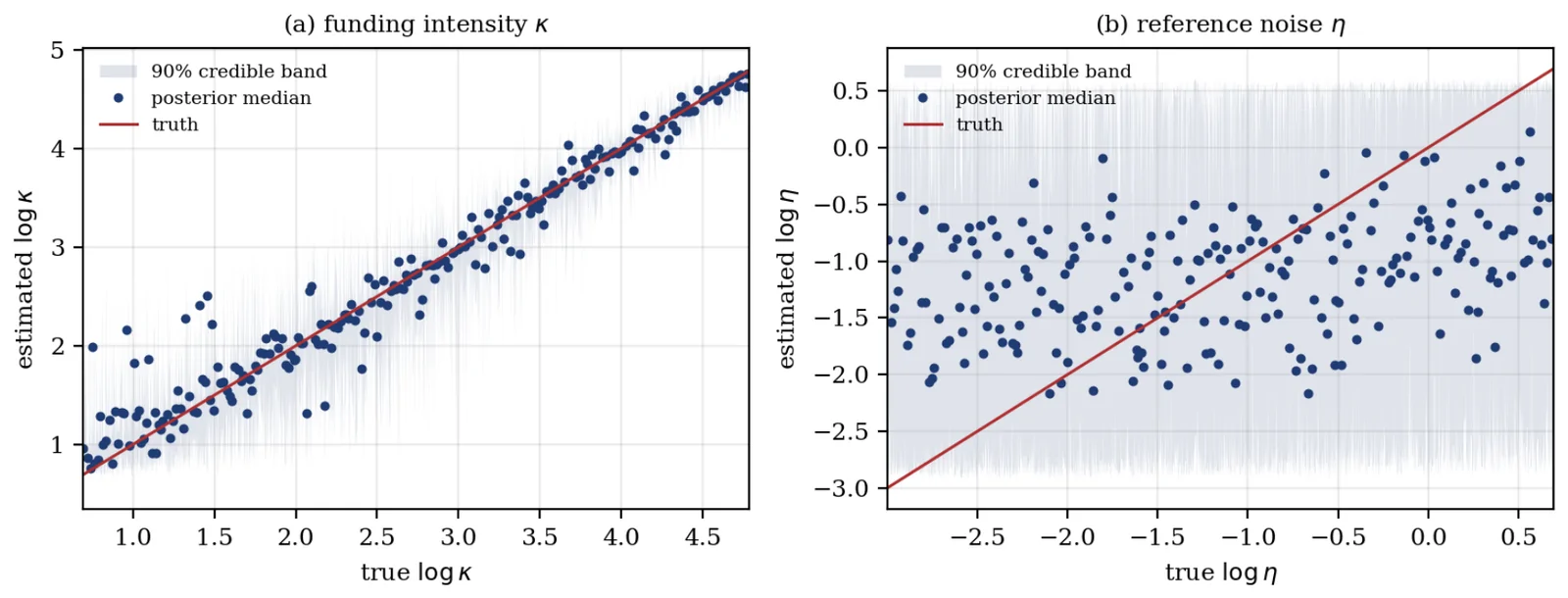
Posterior medians and 90 percent bands against the truth on held-out simulated tapes. The funding intensity is recovered sharply; the reference noise is not, and the intervals correctly report that it is not.

Table 3 and Figure 4 report the outcome, and the pattern is informative rather than uniformly favorable. The funding intensity is sharply identified: the posterior standard deviation is a sixth of the prior and the median tracks the truth with $R^{2}=0.96$. The drift and the fundamental volatility are identified moderately. The reference noise η is barely identified at all, with $R^{2}=0.13$ and a posterior almost as wide as the prior, because η enters the tape only through the filtered index and is there confounded with the price noise $s_{F}$. Coverage remains close to nominal. The weak identification of η bears directly on the paper’s thesis. The softness of the anchor is exactly what η measures, and the experiment says that a venue tape does not reveal it. Assessing how soft an anchor is requires the reference series itself, not the price and funding series it generates. Any empirical program built on this framework should therefore treat η as something to be brought in from outside, through the frequency and dispersion of primary market marks, rather than estimated from traded data.

### 13.3. An Illustrative Calibration

The calibration assigns plausible primitives as of 11 June 2026. Parameters are assumed rather than fitted because the available tape does not identify the reference noise η.

Under Section 8, the perpetual price is the market’s posterior mean for the latent fundamental and the steady-state posterior variance is $P_{\infty }=\sigma \eta$. Taking the 11 June Hyperliquid close of $172.84 as the posterior mean, suppose the primary market mark is refreshed every $T_{r}$ years with a log dispersion of ε, so that $\eta =\varepsilon \sqrt{T_{r}}$. A 90 percent credible interval for the fundamental is then $172.84\exp(\pm 1.645\sqrt{\sigma \eta })$. Table 4 reports the resulting intervals across the grid.

All twelve intervals contain both the $185 secondary close and the $135 offer, so the calibration does not discriminate between them. A soft anchor raises η and therefore $P_{\infty }=\sigma \eta$, producing the wide intervals predicted by Section 8. Narrower intervals require external evidence on the frequency and dispersion of primary market marks. The sharper empirical prediction is the behavior of the basis around the staggered lock-up releases in Section 17.

## 14. The SpaceX Pre-IPO Market: A Worked Example

SpaceX was private with no continuously observable spot and no dividend, so it is the Shiller use case stated literally: an equity claim whose price is difficult or impossible to observe before listing. The instrument the market built for it was the crypto funding variant of Corollary 1(ii), not the dividend variant. Hyperliquid’s xyz:SPCX contract was USD Coin (USDC) settled and Binance’s SPCXUSDT contract was Tether (USDT) margined. Both traded continuously with no expiry and provided price exposure without voting rights, dividends, or a claim on the issuer’s assets. Table 5 collects the event window observations.

**Data.** The event analysis uses UTC daily closing levels. Hyperliquid’s public Info application programming interface (API) supplies 33 xyz:SPCX candles from 17 May through 18 June, 26 before the listing and 7 on or after it. Binance Public Data supplies 29 SPCXUSDT candles from 21 May through 18 June, including the same 7-day post-listing window, together with 85 funding observations at eight-hour intervals. The first retained funding observation is at 16:00 UTC on 21 May, so the first two eight-hour slots are outside the sample. The listed-equity series contains the five Nasdaq sessions from 12 through 18 June. The venues are analyzed separately because their quote assets, funding intervals, and volume fields differ. The extended Hyperliquid sample contains 73 complete days and 1730 hourly candles through 28 July. The hourly closes align with 1729 funding premium observations for the correlation below. The short post-listing window is the principal limitation on the event comparison [17,18,19].

**Figure 5:**
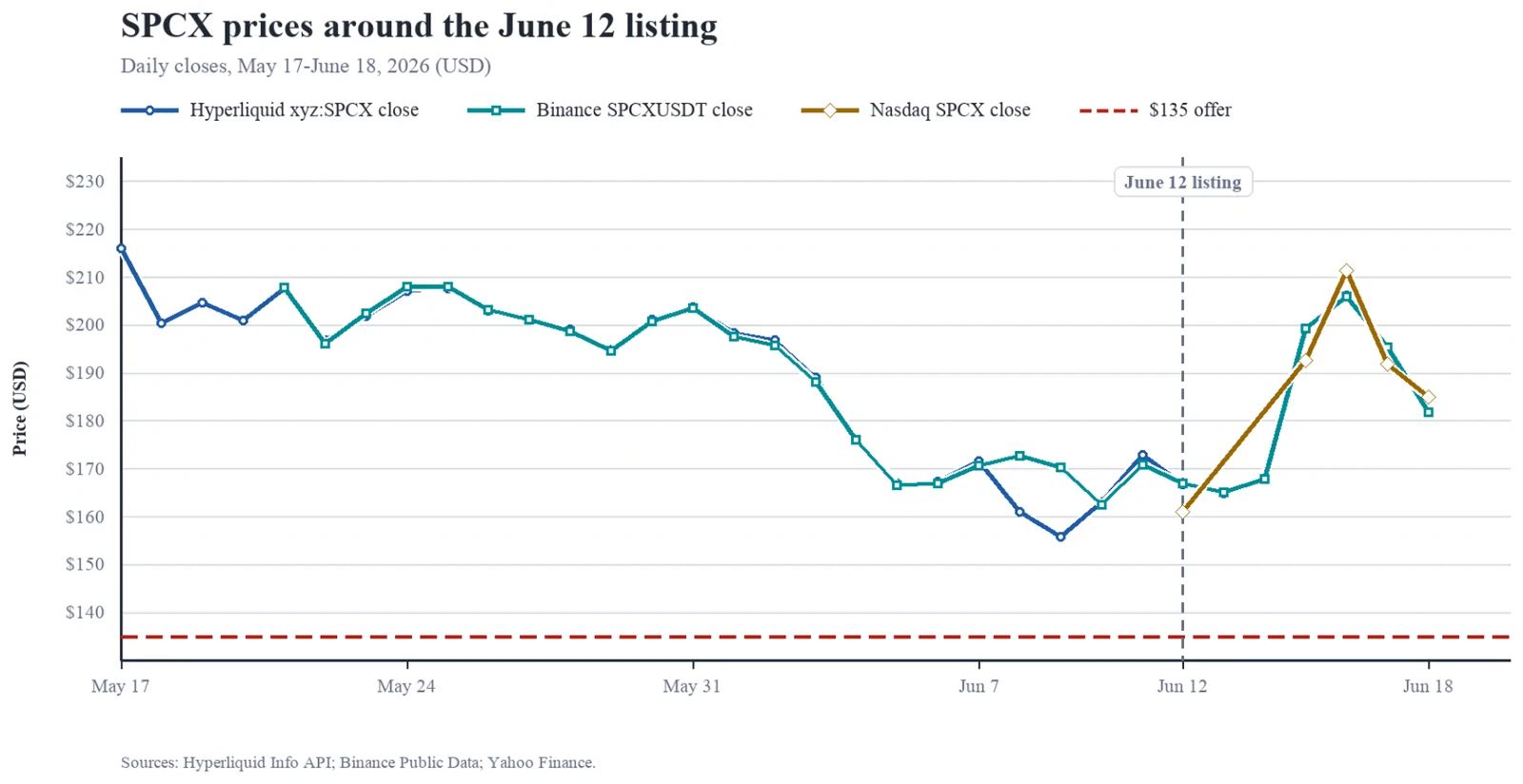
Daily closes for Hyperliquid xyz:SPCX, Binance SPCXUSDT, and Nasdaq SPCX. The vertical line marks the 12 June listing; the horizontal line marks the $135 offer.

**Market activity.** The pre-IPO market was large. On 12 June, Hyperliquid recorded 8.227 million SPCX of base volume and 632,501 trades. Binance SPCXUSDT recorded $5.848 billion of quote volume, ranking third among 787 archived USD-M contracts, behind BTCUSDT at $9.269 billion and ETHUSDT at $6.159 billion. The cumulative SPCXUSDT quote volume from 21 May through 18 June was $22.821 billion. Binance open interest on 12 June ranged from $179.2 million to $325.9 million and peaked at 15:50 UTC. Figure 5 plots the daily closes across the listing.

**The inversion.** The last pre-listing closes of $172.84 on Hyperliquid and $170.82 on Binance were closer to the listed equity’s 18 June close of $185 than the $135 underwritten offer was. Mega-IPO underpricing is well documented [20,21], so this ordering is not by itself surprising: a bookbuilt offer is an administered allocation price, not a forecast of the secondary market. The $135 offer is reported in the issuer’s prospectus [22]. The episode nevertheless shows that a continuously traded contract on a claim with no observable spot produced a level close to where the claim subsequently cleared.

This level comparison is descriptive, not an application of the log-scale Doob martingale in Section 9. In levels, the Jensen correction separates $E[e^{x_{T}}|F_{t}]$ from $e^{E[x_{T}|F_{t}]}$, so no optional stopping equality is asserted here. Against the $185 endpoint, the absolute percentage errors are 6.6 percent for Hyperliquid and 7.7 percent for Binance, compared with 27.0 percent for the offer. These are one-origin, one-event comparisons: they illustrate the pricing framework but do not test forecast superiority or the martingale condition.

**Choice of terminal date.** The $185 endpoint is the listed equity’s 18 June close and is a modeling choice rather than a settled valuation. During its first five Nasdaq sessions, the equity traded between $149.34 and $225.64, so another date in the same week would materially change the comparison. The float also remained restricted. The less date-sensitive prediction is that the perpetual basis should contract as the staggered lock-up releases expand the float.

**Filtering interpretation.** The posterior of Section 8 did not collapse at the offer. Nasdaq closes moved 160.95→192.50→211.39→191.82→185.00 from 12 through 18 June. In the filtering model, these are the first hard observations of the latent value, but five sessions cannot distinguish that interpretation from other processes with the same path. Restricted float keeps the observation noise η and posterior variance $P_{\infty }=\sigma \eta$ elevated; the staggered lock-up releases provide a later test as the float expands.

**Exploratory diagnostics.** Over the 73 complete Hyperliquid daily observations through 28 July, the mean daily log return is −0.00855 (Newey–West standard error 0.00452, p=0.059). The post-listing change in mean absolute funding premium is −0.00746 (Newey–West standard error 0.00577, p=0.196). The correlation between absolute hourly log returns and the absolute funding premium is 0.50, with a 95 percent circular block-bootstrap interval of [0.08,0.72]. These statistics describe within-contract variation; identification of the premium decomposition requires a longer panel.

## 15. Reading the Premium

The pricing sections above are stated under Q. The pre-listing Hyperliquid close of $172.84 against the $135 offer is a statement under the physical measure P with frictions. In the decomposition below, $S_{t}^{\mathrm{IPO}}=135$ is the administered offer price and is the denominator for every log-premium component:

(17) $$\log\frac{F_{t}}{S_{t}^{\;\mathrm{IPO}}}=\underset{\mathrm{risk}\;\mathrm{premium}}{\underset{︸}{\rho_{t}}}+\underset{\mathrm{segmentation}}{\underset{︸}{\zeta_{t}}}+\underset{\mathrm{discovery}}{\underset{︸}{\delta_{t}}}+\underset{\mathrm{bubble}}{\underset{︸}{\upsilon_{t}}}.$$

The risk premium $\rho_{t}$ is the change of measure carried by a leveraged holder of price risk and has an ambiguous sign. The segmentation term $\zeta_{t}$ is the value of the synthetic access of Section 10. $\zeta_{t}\approx \psi_{t}/S_{t}^{\mathrm{IPO}}=\lambda n_{c}/S_{t}^{\mathrm{IPO}}$ for a small premium and is strictly positive. The discovery term $\delta_{t}$ is the gap between the administered offer and the market clearing value, also positive here. The bubble term $\upsilon_{t}$ is the log of a component $\Upsilon_{t}$ for which $\Lambda_{t}\Upsilon_{t}$ is a Q-martingale. It is admissible only if the transversality condition of Proposition 1 fails, and its behavioral counterpart is extrapolative demand.

**Identification.** The identity has four unknowns and one observable, so it is not identified from the price series alone. Any assignment of $\rho_{t},\zeta_{t},\delta_{t}$ determines $\upsilon_{t}$ as a residual. No conclusion about the size of the bubble term follows from (17) without either a prior over the other three components or an external moment condition that pins one of them down.

Fix the discovery term at the gap between the offer and the 18 June listed-equity close, so that $\delta_{t}=\log\left(185/135\right)=0.315$. Using Hyperliquid’s 11 June close, the observed premium is log(172.84/135)=0.247, and the remaining three components therefore sum to −0.068. Repeating the calculation with Binance’s $170.82 close gives −0.080. These are residuals from an identity, not estimates of any individual component. In particular, the discovery term here is not measured but *assigned*, fixed at the offer-to-close gap 0.315, which is large by construction. The small residual −0.068 therefore constrains only the sum $\rho_{t}+\zeta_{t}+\upsilon_{t}$ and says nothing about the magnitude of the bubble term $\upsilon_{t}$ on its own. A small residual is not evidence of a small bubble. A positive segmentation term could be offset by a negative risk premium or bubble term, and the arithmetic alone cannot distinguish those cases. Estimation requires the posterior of Section 11, which places a prior on $\left(\rho_{t},\zeta_{t}\right)$ through the primitives $\gamma_{U}$, $n_{c}$, and $\sigma_{V}$ and returns $\upsilon_{t}$ with a credible interval rather than by subtraction. Both $\delta_{t}$ and the accuracy comparison use the same $185 endpoint and therefore provide no independent corroboration. Moreover, the offer is an administered allocation price and a weak forecasting baseline given documented mega-IPO underpricing.

Subject to those caveats, the sign pattern is what Proposition 6 predicts. A positive segmentation term pins down the ratio $\sigma_{V}^{2}n_{c}/\gamma_{U}$ of constrained demand to arbitrage capacity, and its decay once the spot is hard is the model’s prediction that the basis collapses when borrow and allocation become available. The issuer’s staggered lock-up releases, by enlarging the float and lowering $\zeta_{t}$, provide the natural experiment that would separate the remaining terms.

## 16. Other Applications to Equities

Beyond pre-IPO discovery, the same instrument addresses several long-standing problems:

**Private and pre-IPO price discovery.** The SpaceX mechanism applies to any late-stage private firm. The perpetual consensus is a continuous when-issued price for a claim that otherwise reprices only at funding rounds, and Section 8 describes how informative it is as a function of the reference noise η. The implication for private equity valuation is direct: a fund holding an unlisted position currently marks it between financing rounds by comparable-company methods, which are a soft anchor in the sense of Section 1. A traded perpetual replaces that mark with a price and, more usefully, replaces a point mark with the posterior of Section 8, whose variance is itself a reported quantity. Where such contracts exist, they give a market-based alternative to the stale-price problem that dominates the measurement of private asset returns [23,24].

**Synthetic shorting and the implied borrow rate.** Where stock borrow is expensive or unavailable, the perpetual provides synthetic short exposure, and the funding rate clears the short demand. In the steady state of Proposition 3, the funding rate equals the marginal cost of the arbitrage, so it is a continuous, observable proxy for the securities lending fee which is cleaner than the opaque stock loan market.

**Restricted and locked-up equity.** An employee or pre-IPO holder who cannot yet sell can short the perpetual to hedge, paying the funding carry as the cost of the hedge. This is the concentrated position problem, and it is the live question for SpaceX holders through the staggered lock-up releases.

**Untraded macro and real indices.** Shiller’s original targets include the Case-Shiller home price index, the consumer price index, wages, and GDP. The CPI and GDP are levels rather than flows, so the dividend variant must settle on an observable flow derived from them, such as the measured index change or a specified rate times the level. A perpetual on that flow lets a household hedge human capital, inflation, or housing risk without a transactable spot.

**Perpetual dividend strips.** A perpetual settled on a dividend index is a traded Gordon model. With constant benchmark flow $a_{t}=D$ and funding coefficient $b_{t}=\kappa$, Proposition 1 gives F=D/κ, separating dividend risk from price risk with no expiry and no roll. Under Shiller’s risk-free design, Corollary 1(i) instead gives D/r.

**Roll-free index, factor and variance exposure.** Funding replaces the roll, removing the negative roll yield of calendar futures. A perpetual on realized variance, with funding keyed to the realized variance of the underlying, gives no expiry volatility exposure and is the direct instrument for the stochastic volatility of Section 7.

**Event and when-issued discovery.** The same continuous market prices SPACs, spinoffs, direct listings, and merger targets, where the administered or infrequent price leaves a discovery gap of the kind seen at the SpaceX listing.

**Synthetic asset markets.** Nothing in Proposition 1 requires the benchmark flow to be the flow of a security. Any adapted, observable process $a_{t}$ defines a contract, so the framework covers the synthetic asset markets that settle against indices, model outputs, or oracle feeds. The framework is also a warning about them: by Section 8, the contract inherits the noise of whatever it settles against, and, by Proposition 6, a synthetic without an arbitrageable underlying carries a segmentation premium that is a price of access rather than a mispricing. The design question for such a market is not whether the peg holds but how hard the anchor is.

**Collateral and continuous access.** Because the contract is cash-settled in a stablecoin and trades without interruption, it gives round-the-clock equity exposure outside exchange hours and a collateral-efficient synthetic position for holders who want exposure without custody of the share. The same property that makes it useful, settlement against a reference rather than delivery of the asset, is the source of the soft anchor defined in Section 1 and analyzed in Section 8 and Section 12: the instrument is only ever as well disciplined as the reference it settles against.

## 17. Discussion

**Establishes.** A perpetual contract can manufacture a tradable, continuous price for a private equity claim that has no spot and no dividend, which is the original Shiller goal carried out for equity rather than for homes or wages. The two venues sustained active markets, and their last pre-listing closes sat closer to the selected 18 June equity close than the bookbuilt offer did.

**Does not establish.** By Proposition 3, the peg holds only inside the band $\bar{g}/\kappa$, and, by Section 8, the pre-IPO band is centered on a soft reference rather than a hard spot, so before listing the discipline is to an estimate and not to an arbitrage-enforceable price. By Proposition 6, the observed basis combines a segmentation premium with the gap between the expected listing value and the soft reference; the episode does not identify their separate magnitudes. The pre-IPO level was consistent with the secondary tape, on one path, but the float is still small and the posterior variance still large, so the test of fundamental value as opposed to clearing price is deferred to the staggered lock-up releases. The decomposition of Section 15 is an accounting identity rather than an estimated model, and the size of its bubble term is not identified by the data used here. The residual arithmetic of Section 15 does not conflict with this: there, the discovery term is set by hand to the offer-to-close gap, which is large by construction, so the remaining components sum to a small number. That small sum bounds neither the segmentation term nor the bubble term individually, and, in particular, is not evidence that the bubble term is small. Identifying the separate magnitudes requires the prior-based posterior of Section 11, not the identity.

**Reading.** Shiller’s dividend-settled design prices an unobservable asset by anchoring to a real flow and is self-contained. The crypto funding design anchors to a basis and needs an external reference, which, for a private firm, is soft. By Proposition 4, the two coincide once a hard spot exists, and, by Proposition 2, both are the expected spot at a funding clock. The robust part is the instrument design; the fragile part, made precise by the filtering and segmentation readings, is the quality of the anchor when no hard price exists. The dividend variant that Shiller proposed, settling on a real cash flow, remains largely unbuilt, and is the natural next instrument for the macro and real indices of Section 16.

**Predictions.** The framework is falsifiable. First, by Proposition 6, the basis should collapse toward zero as the float grows and the reference becomes the hard spot, so the perpetual-to-spot premium should fall around the staggered lock-up-release dates. A persistent premium afterward would reject the segmentation reading in favor of a bubble. Second, by Section 16 the funding rate should track the securities lending fee once the stock is borrowable, so the two should converge post-listing. Third, by Corollary 3, the basis is insensitive to the spot’s volatility and responds only to its carry, so a near-martingale equity should peg tightly however volatile it is, while a variance perpetual, whose benchmark is volatility itself, should carry any volatility risk premium. Each is measurable with the data the contracts already generate, and Section 11 gives the estimation route.

**Conclusions.** Shiller’s perpetual future was a proposal to price what cannot be traded. The crypto markets supplied the funding mechanism that made it work at scale, and the SpaceX listing showed that the resulting price can track a private equity claim closely enough to be informative about where it will clear. The unifying object is the funding rate, which is simultaneously a discount rate in Proposition 1, a clock in Proposition 2, an observer gain in Section 8, and a learning rate in Section 9. What remains fragile is the anchor, and the cleanest open instrument is the dividend-settled variant Shiller first described, which needs no anchor at all.

## Figures and Tables

**Table 1:** Corollary 3. Driftless spot, c=0, $S_{0}=100$. The basis is statistically indistinguishable from zero for every funding clock, including one driven by the volatility state and leveraged against the spot.

$\kappa_{0}$	$\kappa_{1}$	ξ	$\rho_{S}$	$F_{0}$	s.e.	*t* for $F_{0}=S_{0}$
12	0	0.50	−0.70	99.9979	0.0179	−0.12
52	0	0.50	−0.70	99.9995	0.0087	−0.06
4	0	0.50	−0.70	99.9943	0.0306	−0.19
12	50	0.50	−0.70	100.0001	0.0139	−0.01
12	50	1.00	−0.90	100.0007	0.0136	−0.05
12	50	1.00	+0.90	99.9993	0.0140	−0.05

**Table 2:** Proposition 5. The basis is exactly the funding-discounted expected carry, and does not depend on the volatility parameters.

*c*	κ	$F_{0}$ (Monte Carlo)	s.e.	$S_{0}\kappa /\left(\kappa -c\right)$	Relative Error
−0.02	12	100.1636	0.0180	100.1669	$-3.3\times 10^{-5}$
−0.05	12	100.4133	0.0180	100.4184	$-5.1\times 10^{-5}$
−0.03	12	99.7502	0.0179	99.7506	$-4.0\times 10^{-6}$
−0.05	52	100.0950	0.0087	100.0962	$-1.2\times 10^{-5}$
−0.10	12	100.8323	0.0182	100.8403	$-8.0\times 10^{-5}$

**Table 3:** Generative Bayesian computation on simulated tapes. Positive parameters are estimated on the log scale. Coverage is the fraction of held-out draws inside the nominal 90 percent band.

	Posterior s.d./Prior s.d.	$R^{2}$ of Median	90% Coverage
drift μ	0.58	0.55	0.89
fundamental vol. σ	0.67	0.49	0.88
reference noise η	0.87	0.13	0.89
funding intensity κ	0.16	0.96	0.87

**Table 4:** Valuation intervals for SpaceX on 11 June 2026 at assumed primitives, anchored at the Hyperliquid close of $172.84. All twelve configurations of the grid σ∈{0.35,0.45,0.55}, ε∈{0.15,0.25}, $T_{r}\in \left\{0.25,0.50\right\}$ are shown.

σ	ε	$T_{r}$	η	$\sqrt{P_{\infty }}$	90% Interval
0.35	0.15	0.25	0.075	0.162	[132.4,225.6]
0.35	0.15	0.50	0.106	0.193	[125.9,237.3]
0.35	0.25	0.25	0.125	0.209	[122.5,243.8]
0.35	0.25	0.50	0.177	0.249	[114.8,260.2]
0.45	0.15	0.25	0.075	0.184	[127.8,233.8]
0.45	0.15	0.50	0.106	0.218	[120.7,247.6]
0.45	0.25	0.25	0.125	0.237	[117.0,255.3]
0.45	0.25	0.50	0.177	0.282	[108.7,274.9]
0.55	0.15	0.25	0.075	0.203	[123.8,241.4]
0.55	0.15	0.50	0.106	0.242	[116.2,257.2]
0.55	0.25	0.25	0.125	0.262	[112.3,266.1]
0.55	0.25	0.50	0.177	0.312	[103.5,288.7]

**Table 5:** SpaceX listing and SPCX event window observations, May to June 2026. Perpetual candles are Coordinated Universal Time (UTC) venue records reported as open/high/low/close (OHLC); equity observations are Nasdaq market data.

UTC Date	Series	Observation
17 May	Hyperliquid	First daily candle: $180.00/$216.00/$179.90/$216.00 (OHLC)
21 May	Binance	First daily candle: $206.00/$224.47/$197.10/$207.76 (OHLC)
11 June	Perpetuals	Last pre-listing closes: Hyperliquid $172.84; Binance $170.82
12 June	Listed equity	$150.00 open; $160.95 close
12 June	Hyperliquid	$172.85/$185.00/$152.34/$166.72 (OHLC)
12 June	Binance	$170.81/$183.76/$152.00/$166.86 (OHLC); $5.848 billion quote volume
16 June	Intraday highs	Listed equity $225.64; Hyperliquid $228.74; Binance $228.00
18 June	Closing levels	Listed equity $185.00; Hyperliquid $181.76; Binance $181.77

## Data Availability

All data supporting the reported results are publicly available. Perpetual futures candles and funding history for the Hyperliquid HIP-3 asset xyz:SPCX were obtained from Hyperliquid’s public Info API [18]; SPCXUSDT klines, funding and metrics were obtained from Binance Public Data [17]; Nasdaq closing prices for SPCX were obtained from Yahoo Finance [19]. The simulation and estimation code used in Section 13 and Section 11 is available from the corresponding author on request.

## Notation

Symbol	Meaning	First used
$f_{t}$	perpetual price, discrete time	Section 2
$F_{t}$	perpetual price, continuous time	Section 3
$S_{t}$	spot or reference level of the underlying	Section 3
$\beta_{t}=F_{t}-S_{t}$	basis	Section 3
$D_{t}$, $q_{t}$	dividend or rent flow; yield when $D_{t}=q_{t}S_{t}$	Section 2
$r_{t}$	one-period risk-free interest rate	Section 2
Q, P	risk-neutral and physical probability measures	Section 1
$\Sigma_{t}$, $\Delta \Sigma_{t}$	cumulative and per-period settlement	Section 2
$a_{t}$	benchmark flow in the settlement rule	Section 3
$b_{t}$	settlement rate coefficient	Section 3
κ	funding intensity, the case $b_{t}=\kappa$	Corollary 1
$G_{t}=F_{t}+\Sigma_{t}$	cumulative gain, a Q-martingale	Section 3
$\Lambda_{s}$, $\Lambda_{s}^{r}$, $A_{s}$	discount factors at *b* and *r*; $A_{s}=\int_{0}^{s}b_{u}du$	Proposition 1
τ	first event of the funding clock	Proposition 2
$g_{t}$, $\bar{g}$	funding payment and its cap	Section 5
χ	carry cost of the basis trade	Section 5
$c_{t}$	carry of the underlying under Q	Section 7
$v_{t}$, ξ, $\rho_{S}$	variance state, vol of vol, leverage	Section 7
$\kappa_{v}$, $\kappa_{0}$, $\kappa_{1}$	variance and funding-clock coefficients	Section 13
$x_{t}$	latent log fundamental	Section 8
$y_{t}$, η	observation process and its noise	Section 8
${\hat{x}}_{t}$, $P_{t}$	filtered estimate and posterior variance	Section 8
μ, σ, ϕ	drift, volatility, mean reversion of *x*	Section 8
$K_{\infty }$	steady state observer gain	Section 8
$p_{t}=E\left[x_{T}|F_{t}\right]$	price as predictor of the listing value	Section 9
$\alpha_{k}$	stochastic approximation step size	Section 9
$n_{c}$, $\gamma_{U}$	constrained demand, unconstrained risk tolerance	Section 10
$\psi_{t}$, $\omega_{t}$	segmentation premium and reference gap	Section 10
$\tilde{V}$, $\sigma_{V}$	listing value and its dispersion	Proposition 6
λ	price per unit of short exposure	Proposition 6
Θ, z	parameter vector and summary statistics	Section 11
$s_{F}$	nuisance price-noise scale	Section 13.2
$M_{t}$, $I_{t}$, ι, $\bar{r}$	mark, index, fixed rate, premium-rate cap	Section 12
*h*, $N_{t}$	funding interval and position notional	Section 12
ε, $T_{r}$	primary-mark dispersion and refresh interval	Section 13
$\rho_{t},\zeta_{t},\delta_{t},\upsilon_{t}$	risk premium, segmentation, discovery, bubble	Section 15
